# Data-Driven Operational Bounds of Transmembrane Pressure for Modelling and Digital Twin Development in Haemodialysis and Haemodiafiltration

**DOI:** 10.3390/bioengineering13030331

**Published:** 2026-03-12

**Authors:** Alexandru Dinu, Mădălin Frunzete, Denis Mihailovschi

**Affiliations:** Faculty of Electronics, Telecommunications and Information Technology, National University of Science and Technology Politehnica Bucharest, 061071 Bucharest, Romania; alexandru.dinu2408@upb.ro (A.D.); denis.mihailovschi@stud.fim.upb.ro (D.M.)

**Keywords:** haemodialysis, haemodiafiltration, transmembrane pressure, operational modelling, data-driven analysis, biomedical engineering

## Abstract

Transmembrane pressure (TMP) is a central state variable in haemodialysis (HD) and haemodiafiltration (HDF), governing ultrafiltration dynamics, convective transport, and membrane performance. Although dialysis devices specify high maximum allowable pressure limits derived from in vitro testing and mechanical safety margins, the effective operating pressure space encountered under routine clinical conditions remains insufficiently quantified from a systems engineering perspective. In this study, aggregated real-world minimum–maximum TMP intervals collected from four geographically distributed dialysis centres were used to anchor a model-based characterisation of operational pressure ranges. To enable reproducible modelling and numerical exploration, Gaussian-based synthetic datasets were constructed from empirically observed pressure intervals while incorporating physiological and operational constraints. Across all centres, HD exhibited stable and narrowly distributed TMP values (typically 20–60 mmHg), whereas HDF operated within higher but well-defined pressure regimes (approximately 120–260 mmHg). Values above 300 mmHg were rare, and pressures exceeding 400 mmHg were not observed under routine conditions. Statistical tail modelling, extreme value theory, and unsupervised anomaly detection consistently identified such extreme pressures as structurally incompatible with the learned operational state space. These results provide quantitative engineering bounds for TMP that may be directly integrated into reduced-order models, control design, and digital twin development for dialysis systems. By constraining modelling environments to empirically supported pressure regimes, the proposed framework enhances numerical stability, prevents non-physical extrapolation, and supports physiologically realistic data-driven applications in biomedical engineering.

## 1. Introduction

Haemodialysis (HD) and haemodiafiltration (HDF) are extracorporeal renal replacement therapies designed to substitute key kidney functions through controlled solute and fluid exchange across semi-permeable membranes. Solute transport is governed by diffusion, driven by concentration gradients and convection, and also driven by hydrostatic pressure gradients across the membrane. In high-volume convective modalities such as HDF, transmembrane pressure (TMP) becomes a primary operational variable, directly influencing ultrafiltration rate, filtration fraction, membrane loading, and overall treatment efficiency [1]. Modulation of TMP has been shown to affect not only clearance performance but also protein loss and membrane stress, highlighting its dual physiological and engineering relevance.

TMP represents the net hydrostatic pressure difference between the blood and dialysate compartments across the dialysis membrane. In clinical practice, it is continuously monitored as a surrogate marker of membrane performance, haemoconcentration, and extracorporeal circuit stability. However, TMP is not directly measured within the hollow fibres of the dialyser; instead, it is estimated from pressure measurements acquired at selected locations along the extracorporeal circuit. Variations in sensor placement, calibration, and calculation methodology may therefore influence the reported TMP values and complicate cross-device or cross-centre comparisons [2]. This measurement dependency underscores the importance of interpreting TMP as a derived engineering parameter rather than a directly observed physical quantity.

Clinical practice guidelines recognise the importance of maintaining stable haemodynamic and pressure conditions during dialysis sessions [3]. At the same time, the dynamic interaction between TMP, ultrafiltration coefficient, and infusion rate in HDF remains complex. Progressive membrane fouling, formation of secondary protein layers, and changes in blood viscosity alter hydraulic permeability over time, thereby modifying the relationship between imposed ultrafiltration rates and the resulting pressure gradients [4]. Consequently, TMP cannot be considered a static control variable but rather a dynamically evolving system state.

From a device engineering standpoint, modern dialysis systems are constructed with substantial mechanical safety margins. Manufacturer specifications frequently report high maximum allowable TMP limits, often derived from controlled in vitro testing scenarios designed to ensure structural integrity and compliance with safety standards [5]. In parallel, research efforts aimed at developing hybrid extracorporeal support platforms—such as combined lung–kidney assist devices employing alternative flow configurations—operate within experimentally defined pressure envelopes that may differ substantially from routine clinical practice [6]. These upper bounds primarily define structural tolerance and fail-safe conditions, rather than realistic in vivo operating regimes.

The distinction between mechanical safety limits and effective operational ranges becomes particularly relevant in the context of biomedical modelling and control. Data-driven approaches have demonstrated that statistically consistent boundary estimation and structural consistency analysis can delineate feasible state spaces in complex systems [7]. Similar modelling principles have been applied in nonlinear dynamical systems to characterise admissible operating regions and exclude non-physical solutions [8]. Moreover, in AI-assisted modelling environments, constraining training data to empirically supported regimes improves robustness, reduces extrapolation artefacts, and enhances interpretability [9]. Translating these principles to dialysis engineering requires a clear definition of the TMP ranges that are actually encountered during routine treatments.

Recent clinical investigations further emphasise the importance of haemodynamic stability and controlled pressure conditions for optimising dialysis dose, electrolyte balance, and fluid removal [10]. Additionally, membrane hemocompatibility and dialysis practice patterns influence both physiological responses and patient-reported outcomes, reinforcing the need to operate within stable and tolerable pressure windows [11]. Emerging modalities such as intermittent infusion haemodiafiltration introduce additional complexity in pressure dynamics, further highlighting the necessity of quantitatively characterising realistic TMP behaviour under varying treatment strategies [12,13,14,15].

Despite the central operational and engineering relevance of TMP, systematic evidence describing the effective pressure space encountered in routine HD and HDF across multiple centres remains limited [16,17]. A rigorous and empirically anchored modelling framework for defining operational TMP bounds is therefore required to bridge the gap between mechanical safety specifications and clinically attainable operating states. Such quantification is essential for the development of physiologically realistic reduced-order models [18,19,20,21], digital twins of dialysis systems, and data-driven optimisation frameworks that avoid reliance on non-physical extreme pressure assumptions.

The directional difference between HD and HDF pressure regimes has been physiologically established [1,4,5]; however, no prior work has provided a probabilistic framework that quantifies exceedance probabilities for extreme TMP thresholds, characterises upper-tail behaviour through extreme value analysis, or defines a learned operational state space suitable for digital twin parametrisation. Translating established clinical knowledge into engineering-usable numerical bounds with associated statistical support remains an open problem in dialysis system modelling.

The aim of this study is to quantitatively characterise the operational transmembrane pressure ranges encountered during routine haemodialysis and haemodiafiltration across four geographically distributed dialysis centres, to construct a statistically supported representation of the realistic TMP operating space using Gaussian-based synthetic datasets anchored in empirically observed intervals, and to evaluate the plausibility of extreme TMP values through tail probability estimation, extreme value theory, and anomaly detection— thereby providing engineering bounds suitable for reduced-order modelling and digital twin development in dialysis systems.

## 2. Materials and Methods

### 2.1. Clinical Data

Clinically observed transmembrane pressure (TMP) ranges were collected during routine dialysis treatments in four geographically distributed dialysis centres in Romania, anonymised as Location 1 to Location 4. Each centre provided data in aggregated form, based on approximately 20 routine TMP measurements per centre and per treatment modality. Data were stratified by haemodialysis (HD) and haemodiafiltration (HDF). No individual patient-level measurements or time-resolved treatment trajectories were accessed.

For each centre and modality, the reported minimum and maximum TMP values were used to characterise the operational pressure interval observed under routine conditions. Given that only aggregated minimum–maximum values from relatively small samples were available, direct estimation of variance was not feasible.

Assuming approximately Gaussian behaviour during stable treatment phases, the expected statistical range of a sample of about 20 observations typically spans roughly 3–4 standard deviations of the underlying distribution. To avoid underestimating variability while acknowledging the limited sample size, the observed clinical interval was conservatively interpreted as corresponding to approximately three standard deviations of the underlying distribution. Accordingly, the standard deviation was estimated as

$$\sigma \approx \frac{{\mathrm{TMP}}_{\max}-{\mathrm{TMP}}_{\min}}{3}.$$

This approximation should be interpreted as a pragmatic modelling choice rather than a statistically exact estimator of dispersion. Given the limited sample size and the availability of only aggregated minimum–maximum information, the resulting parametrisation is intended to support exploratory modelling and sensitivity analysis rather than precise inference on the true empirical distribution.

The mean TMP value for each centre and modality was approximated as the midpoint of the reported interval. These parameters were used exclusively for descriptive comparison and subsequent modelling purposes and do not represent direct measurements of individual patient-level variability.

In Table 1, each row represents one centre–modality pair. For example, the first entry (L1, HD) indicates that, in Location 1, approximately 20 routine haemodialysis sessions yielded TMP values between 25 and 55 mmHg, with an estimated mean of 40 mmHg obtained as the midpoint of the interval. The corresponding HDF entry reflects the operational TMP interval observed under haemodiafiltration in the same centre.

Across all centres, TMP values during routine haemodialysis remained consistently low, with mean values between 40 and 50 mmHg and relatively narrow estimated dispersion. In contrast, haemodiafiltration was associated with systematically higher pressure ranges, with mean TMP values between 180 and 215 mmHg, reflecting increased convective requirements.

Importantly, within the approximately 20 routine observations available per centre and modality, no TMP values exceeding 300 mmHg were reported during routine operation. The clear and reproducible separation between HD and HDF pressure regimes across geographically distinct centres suggests stable operational behaviour under real-world clinical conditions. These empirically observed intervals form the basis for the subsequent statistical modelling and synthetic data generation.

### 2.2. Synthetic Data Generation

To enable extended statistical analysis and exploratory data-driven modelling beyond the limitations imposed by aggregated clinical observations, a synthetic dataset of transmembrane pressure (TMP) values was generated. The construction of the synthetic data was anchored in the empirically observed TMP ranges and central tendencies reported across the participating dialysis centres, while remaining consistent with the physiological and operational constraints described in the literature.

For each dialysis modality (haemodialysis and haemodiafiltration) and centre, the available clinical information consisted of aggregated minimum–maximum TMP values derived from approximately 20 routine measurements. Given the limited sample size (n≈20), the observed range cannot be assumed to represent the full population spread or the theoretical ±3σ limits of a normal distribution.

Building on the dispersion approximation introduced in Section 2.1, the clinical interval was conservatively interpreted as spanning approximately three standard deviations of the underlying process, while the mean value μ was taken as the midpoint of the reported interval.

This parametrisation intentionally yields a dispersion slightly broader than the empirically observed interval in order to avoid artificially constraining the operational state space and to ensure that potential near-boundary behaviours are not suppressed by modelling assumptions.

Synthetic TMP values were subsequently generated by sampling from Gaussian distributions parameterised by the estimated μ and σ, without artificially truncating the distribution to the observed clinical range. This approach preserves a non-zero probability of generating values outside the reported interval, allowing us to assess whether substantially higher TMP values could plausibly emerge from the empirically supported variability, rather than being imposed or excluded by construction.

The role of the synthetic dataset was strictly methodological. Synthetic data were used exclusively to support statistical exploration, sensitivity analysis, and feasibility assessments for modelling and machine learning applications under clinically realistic operating conditions. No clinical conclusions were derived from the synthetic data, and the dataset does not represent individual patient-level trajectories or real treatment outcomes.

**Figure 1:**
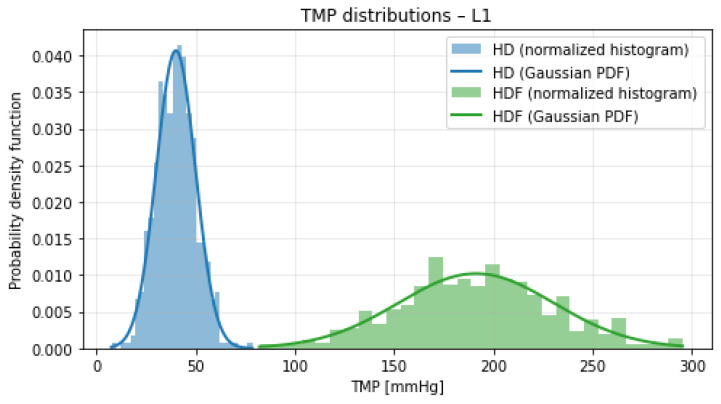
Normalized histograms of synthetic TMP distributions for haemodialysis (HD, blue) and haemodiafiltration (HDF, green) at Location 1. Solid lines indicate the corresponding theoretical Gaussian probability density functions.

**Figure 2:**
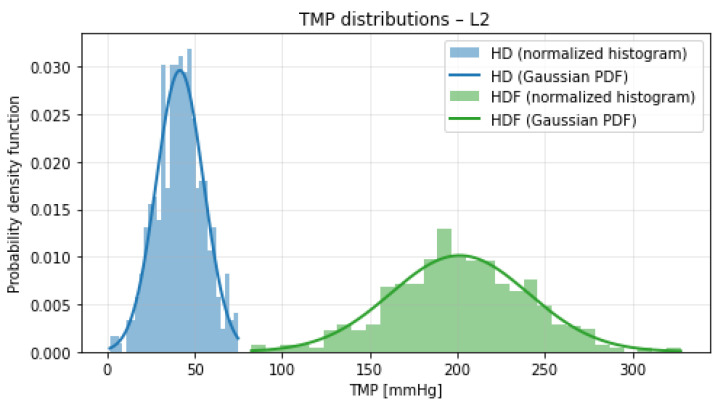
Normalized histograms of synthetic TMP distributions for haemodialysis (HD, blue) and haemodiafiltration (HDF, green) at Location 2. Solid lines indicate the corresponding theoretical Gaussian probability density functions.

**Figure 3:**
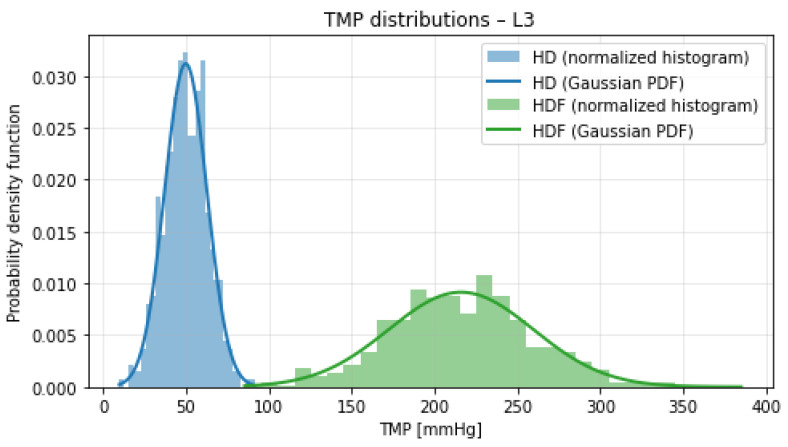
Normalized histograms of synthetic TMP distributions for haemodialysis (HD, blue) and haemodiafiltration (HDF, green) at Location 3. Solid lines indicate the corresponding theoretical Gaussian probability density functions.

**Figure 4:**
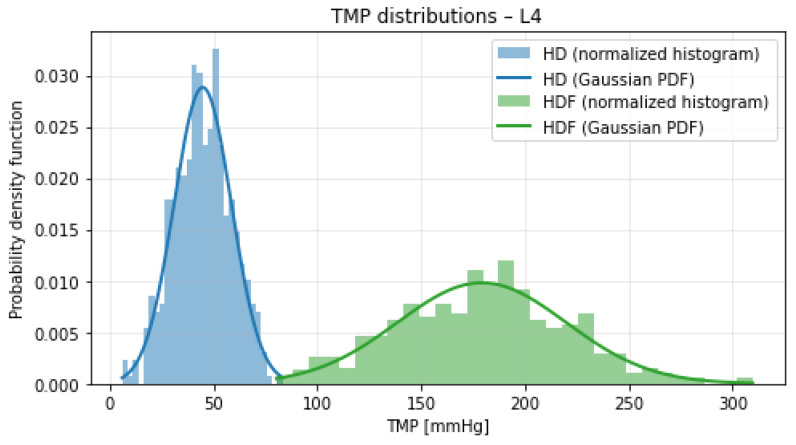
Normalized histograms of synthetic TMP distributions for haemodialysis (HD, blue) and haemodiafiltration (HDF, green) at Location 4. Solid lines indicate the corresponding theoretical Gaussian probability density functions.

Figure 1, Figure 2, Figure 3 and Figure 4 illustrate the normalized histograms of synthetic transmembrane pressure (TMP) values for haemodialysis (HD) and haemodiafiltration (HDF) across the four participating dialysis centres. The synthetic distributions were generated using centre-specific mean values and dispersion parameters derived from the clinically observed intervals under the assumption that the reported range corresponds to approximately three standard deviations of the underlying process.

Across all locations, HD treatments are characterised by comparatively low TMP values and concentrated distributions centred around 40–50 mmHg. Although the conservative dispersion assumption (range ≈3σ) produces moderately wider tails than a strictly data-matching variance estimate, this choice prevents underestimation of variability and maintains modelling robustness near operational boundaries.

In contrast, HDF consistently exhibits higher mean TMP levels (approximately 180–215 mmHg) together with visibly broader distributions. The increased variance reflects the convective nature of haemodiafiltration and the higher hydraulic loads required to sustain substitution volumes. The Gaussian overlays show close agreement with the simulated histograms, indicating that the parametrisation is internally consistent. Despite the increased dispersion introduced by the 3σ-based modelling assumption, a clear and reproducible separation between HD and HDF pressure regimes is maintained across all centres. The overlap between the two distributions remains minimal, and no systematic centre-specific shift toward extreme TMP values is observed.

**Figure 5:**
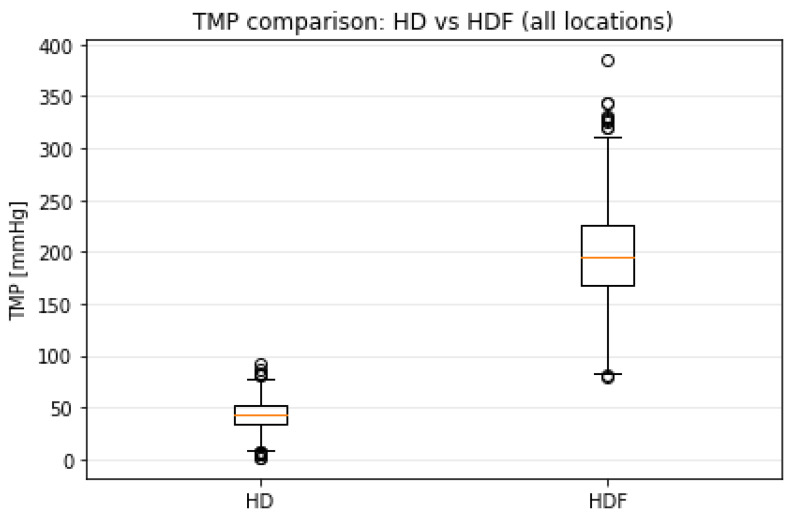
Boxplot comparison of synthetic transmembrane pressure (TMP) values for haemodialysis (HD) and haemodiafiltration (HDF) across all locations.

The global comparison presented in Figure 5 further highlights the structural distinction between modalities. Median TMP values for HDF are substantially higher than those observed during HD, and the interquartile ranges are correspondingly wider. While the broader variance assumption allows occasional synthetic values above 300 mmHg to appear in the upper tails, such occurrences remain infrequent relative to the core operating region.

Overall, the synthetic distributions reproduce the clinically observed separation between HD and HDF while preserving a realistic level of dispersion consistent with the limited number of measurements used to estimate variability.

### 2.3. Statistical Plausibility of Extreme TMP Values

To rigorously assess the plausibility of extreme transmembrane pressure (TMP) values under the simulated operational regimes, three complementary analytical approaches were employed: (i) Gaussian tail probability estimation, (ii) Extreme Value Theory (EVT) modelling of upper-tail behaviour, and (iii) distribution-free anomaly detection using Isolation Forest. Each method captures a distinct structural aspect of the TMP distributions and provides complementary structural insight into the inferred operational bounds.

Table 2 reports exceedance probabilities for selected TMP thresholds. The columns *Mean* and *StdDev* denote the estimated parameters of the synthetic Gaussian distributions for each dialysis modality. The quantity $P_{\mathrm{Gaussian}}$ represents the analytical upper-tail probability P(TMP>T) under the fitted normal model, while $P_{\mathrm{Empirical}}$ corresponds to the observed proportion of simulated values exceeding the specified threshold.

For haemodialysis (HD), the estimated mean is 44.03 mmHg with standard deviation 13.12 mmHg. All investigated thresholds (300–600 mmHg) lie more than 19 standard deviations above the mean. Consequently, both analytical and empirical exceedance probabilities are numerically zero, confirming that such pressures are structurally incompatible with the HD operational regime.

For haemodiafiltration (HDF), the estimated mean is 196.96 mmHg with standard deviation 42.89 mmHg. A threshold of 300 mmHg corresponds to approximately 2.4 standard deviations above the mean, yielding a Gaussian exceedance probability of $8.14\times 10^{-3}$ (0.8%), which is consistent with the empirical frequency of 1%. At 400 mmHg (approximately 4.7 standard deviations above the mean), the exceedance probability drops to $1.10\times 10^{-6}$, with no empirical occurrences observed in the simulated dataset. For 500 mmHg and 600 mmHg, theoretical probabilities become negligibly small, confirming that such values lie in the extreme asymptotic tail of the distribution.

Table 3 summarises the results of the Extreme Value Theory analysis. The threshold $u_{95}$ corresponds to the empirical 95th percentile used for peak-over-threshold modelling. Exceedances above $u_{95}$ were fitted with a Generalized Pareto Distribution (GPD). The parameter ξ denotes the shape coefficient controlling tail behaviour, while β represents the scale parameter.

For HD, the shape parameter ξ=−0.096 is negative, indicating a bounded upper tail and finite support. Exceedance probabilities beyond 300 mmHg are effectively zero, confirming rapid tail decay.

For HDF, the shape parameter ξ=−0.020 remains negative but closer to zero, indicating a finite yet less sharply bounded upper tail compared with HD. The EVT-based probability for 300 mmHg is $1.01\times 10^{-2}$, consistent with Gaussian and empirical estimates. For 400 mmHg and above, EVT probabilities decay rapidly from $10^{-5}$ to $10^{-10}$, confirming that these thresholds lie in the asymptotic extreme region of the operational distribution.

Negative ξ values in both modalities indicate bounded tail behaviour within the simulated pressure regimes and are consistent with the absence of heavy-tailed amplification effects.

Table 4 presents anomaly detection results obtained using Isolation Forest. The anomaly score reflects the average path length required to isolate a test value within randomly partitioned trees. In the present one-dimensional setting, Isolation Forest serves primarily as a distribution-free structural consistency check, complementing parametric tail modelling rather than replacing it. More negative scores indicate stronger deviation from the learned operational regime. The label −1 denotes classification as an anomaly.

For HD, all thresholds between 300 and 600 mmHg receive identical anomaly scores (−0.270), reflecting extreme isolation relative to the tightly bounded HD distribution.

For HDF, the anomaly score at 300 mmHg (−0.179) is less negative than for higher thresholds, indicating that 300 mmHg remains closer to the distribution boundary. At 400 mmHg and above, scores decrease to approximately −0.289, reflecting strong structural incompatibility with the learned operational state space.

The convergence of parametric modelling, asymptotic tail analysis, and distribution-free anomaly detection provides robust confirmation that TMP values above 400 mmHg are statistically implausible within routine operational variability, while 300 mmHg represents an extreme yet statistically admissible level in haemodiafiltration.

## 3. Results

Across the four participating dialysis centres, the aggregated clinical data reveal consistent operational TMP intervals: HD ranged from 20–70 mmHg across centres (midpoints 40–50 mmHg), while HDF ranged from 120–280 mmHg (midpoints 180–215 mmHg). No values exceeding 300 mmHg were reported under routine conditions at any centre. Inter-centre variability was modest, with HD midpoints spanning 10 mmHg and HDF midpoints spanning 35 mmHg, indicating broadly consistent operational practices across geographically distributed sites.

Across the four participating dialysis centres, synthetic distributions constructed from aggregated clinically observed intervals yield model-based mean TMP values of approximately 44 mmHg for haemodialysis and 197 mmHg for haemodiafiltration. Haemodialysis exhibits a narrow dispersion (standard deviation 13.12 mmHg), whereas haemodiafiltration demonstrates broader variability (standard deviation 42.89 mmHg), reflecting increased convective demands. These model-based parameters are consistent with the empirically observed midpoints reported above.

Gaussian, EVT, and anomaly detection analyses consistently demonstrate that:

- Haemodialysis operates within a tightly bounded pressure regime with negligible probability of exceeding 300 mmHg.
- Haemodiafiltration admits a wider operational envelope, with approximately 1% model-based probability of exceeding 300 mmHg. Given the limited empirical sample size (approximately 20 observations per centre), the absence of such values in the aggregated clinical data remains statistically consistent with this low exceedance probability.
- Pressures of 400 mmHg and above lie in the asymptotic extreme tail of the HDF distribution.
- EVT modelling confirms finite upper support for both modalities.
- Isolation Forest classifies all thresholds above 300 mmHg as structural anomalies within the learned operational state space.

These analyses collectively define the following engineering bounds for TMP in dialysis system modelling and digital twin development:

- HD modelling envelope: the empirically supported range is 20–70 mmHg; values above 150 mmHg should be treated as non-physical in HD modelling contexts.
- HDF modelling envelope: the empirically supported range is 120–280 mmHg; 300 mmHg represents the practical upper boundary for digital twin parametrisation and should trigger anomaly flags rather than being accommodated as a normal operating state.
- Exclusion threshold: values above 400 mmHg are statistically and structurally incompatible with routine operation under the present modelling framework for both modalities.

## 4. Discussion

The model-based statistical analysis performed in this study provides a quantitative framework for interpreting extreme transmembrane pressure (TMP) levels in relation to empirically observed operational regimes. Under the conservative 3σ variability assumption adopted in the present paper, a threshold of 300 mmHg represents an extreme yet statistically admissible level within the model-based distribution of haemodiafiltration pressures, even though it was not observed in the limited aggregated empirical sample.

It should be emphasised that the adopted 3σ interpretation of the observed clinical interval intentionally yields a slightly broader dispersion than a direct data-matching estimate would provide. This conservative modelling choice ensures that the inferred operational bounds are not artificially tightened by limited sample size and that potential near-boundary behaviours remain statistically representable within the synthetic framework.

In contrast, thresholds above 400 mmHg lie deep in the asymptotic tail of the distribution. Both Gaussian modelling and extreme value theory indicate rapidly vanishing exceedance probabilities, while Isolation Forest classifies such values as structural anomalies. The negative EVT shape parameters further confirm bounded upper tails and exclude heavy-tailed amplification effects. In practical terms, TMP levels of 400–600 mmHg are not merely rare; they are statistically incompatible with routine operational variability.

From an engineering standpoint, this distinction is critical. Device specifications sometimes report maximum allowable TMP limits in the range of 500–600 mmHg, typically derived from in vitro mechanical stress testing and worst-case safety scenarios. However, the present analysis demonstrates that such limits are not representative of statistically supported in vivo operating states. A threshold of 500 mmHg is as improbable in routine clinical practice as 600 mmHg, with both lying well beyond the empirical and asymptotic bounds of observed behaviour.

These findings are broadly consistent with pressure levels reported in the clinical dialysis literature. Pedrini et al. [1] reported TMP values in the range of 150–250 mmHg during high-volume mixed haemodiafiltration, while Lang et al. [5] described operational pressure envelopes consistent with the HDF ranges identified in the present analysis. For haemodialysis, the low and narrowly distributed TMP values observed across all four centres are in agreement with the stable haemodynamic profiles reported by Stuard et al. [10] under routine HD conditions. Importantly, none of these clinical sources report sustained TMP values above 300 mmHg under stable treatment conditions, which is consistent with the statistical characterisation presented here. The present work extends these clinical observations by providing a probabilistic quantification of the operational pressure space and an explicit evaluation of extreme pressure plausibility within a modelling framework.

Importantly, in real-world operation, control systems, alarm mechanisms, and flow regulation strategies would intervene before sustained exposure to such extreme pressures could occur. Consequently, the probability of reaching 500–600 mmHg under stable treatment conditions is not only statistically negligible but operationally implausible. In this sense, very high specification limits represent mechanical safety margins rather than clinically meaningful or physiologically representative working ranges.

The consistent structural indications obtained from parametric modelling, extreme value analysis, and distribution-free anomaly detection strengthen the internal coherence of this interpretation within the adopted modelling framework and align with established approaches for structural boundary detection in complex systems [7,8,9].

Several limitations of the present study should be acknowledged. First, the clinical information available from each dialysis centre consisted of aggregated minimum–maximum TMP values derived from approximately twenty routine observations per centre and modality. Observed minimum–maximum ranges based on such small samples are sensitive to occasional outliers: a single atypical session could shift the reported interval by 10–20 mmHg, which under the adopted 3σ approximation would correspondingly alter the estimated dispersion and tail probabilities. This sensitivity represents an inherent limitation of the available data and further supports interpreting the results as exploratory rather than inferential. Second, the statistical exploration relied on synthetic data generation under a Gaussian modelling assumption. Although this enables methodological analysis of plausible operating regimes, the synthetic data do not represent individual patient-level trajectories and should be interpreted as a modelling framework rather than direct clinical evidence. Third, the aggregated dataset did not include prescription-level variables such as blood flow rate, ultrafiltration rate, substitution volume, filtration fraction, dialyser type, or blood-side properties. The resulting bounds therefore reflect the integrated effect of all operational variables under standard practice conditions at the participating centres, which represents the relevant reference frame for digital twin parametrisation but precludes covariate-specific analysis. Non-routine events such as circuit clotting, line occlusion, or transient flow disturbances may produce temporary pressure excursions beyond these operational bounds and were not considered within the scope of the present modelling framework.

Beyond modelling applications, these results may support clinical practice by providing a realistic reference range for transmembrane pressure behaviour during routine dialysis treatments. Specifically, the quantitative bounds derived here suggest that sustained TMP values above 300 mmHg during haemodiafiltration, while statistically rare, warrant technical review, whereas values approaching or exceeding 400 mmHg are inconsistent with stable operational conditions and should prompt immediate circuit assessment. For haemodialysis, the analysis supports maintaining awareness that even values above 150 mmHg represent a substantial deviation from the routine operational regime and may reflect early circuit dysfunction or measurement anomaly.

These findings also carry direct implications for modelling, device evaluation, and digital twin development. Constraining simulation environments and algorithmic control frameworks to empirically validated TMP bounds enhances numerical stability, avoids non-physical extrapolation, and supports physiologically realistic parameterisation. More broadly, distinguishing clearly between mechanical stress tolerances and clinically attainable pressure regimes improves the interpretability of technical documentation and prevents overestimation of operational risk envelopes.

Future work may expand upon the present framework by incorporating larger multi-centre datasets and time-resolved TMP measurements collected directly during dialysis sessions. Such data would enable direct estimation of distributional properties and allow integration of patient-specific and treatment-specific variables into predictive modelling. In addition, embedding empirically validated pressure bounds into real-time monitoring systems and digital twin platforms could support adaptive control strategies and further improve the safety and efficiency of advanced dialysis therapies.

In summary, while high TMP specification limits ensure structural safety, they are statistically and operationally non-representative of routine dialysis practice. A data-driven definition of realistic TMP bounds provides a more meaningful reference framework for clinical optimisation, device comparison, and advanced modelling applications.

## Figures and Tables

**Table 1:** Clinically observed TMP ranges and mean values (mmHg) during routine dialysis.

Site	Mode	Range	Mean
L1	HD	25–55	40
L1	HDF	130–250	190
L2	HD	20–60	40
L2	HDF	140–260	200
L3	HD	30–70	50
L3	HDF	150–280	215
L4	HD	25–65	45
L4	HDF	120–240	180

**Table 2:** Tail probability estimation for extreme transmembrane pressure thresholds (values expressed in mmHg).

Mode	Threshold	Mean	StdDev	$P_{\mathrm{Gaussian}}$	$P_{\mathrm{Empirical}}$
HD	300	44.03	13.12	0	0
HD	400	44.03	13.12	0	0
HD	500	44.03	13.12	0	0
HD	600	44.03	13.12	0	0
HDF	300	196.96	42.89	$8.14\times 10^{-3}$	0.01
HDF	400	196.96	42.89	$1.10\times 10^{-6}$	0
HDF	500	196.96	42.89	$8.00\times 10^{-13}$	0
HDF	600	196.96	42.89	0	0

**Table 3:** Extreme value analysis of transmembrane pressure using a Generalized Pareto Distribution fitted to exceedances above the 95th percentile (values expressed in mmHg).

Mode	Threshold	$u_{95}$	Shape ξ	Scale β	$P_{\mathrm{EVT}}$
HD	300	66.75	−0.096	5.29	0
HD	400	66.75	−0.096	5.29	0
HD	500	66.75	−0.096	5.29	0
HD	600	66.75	−0.096	5.29	0
HDF	300	267.81	−0.020	20.39	$1.01\times 10^{-2}$
HDF	400	267.81	−0.020	20.39	$4.86\times 10^{-5}$
HDF	500	267.81	−0.020	20.39	$1.26\times 10^{-7}$
HDF	600	267.81	−0.020	20.39	$1.47\times 10^{-10}$

**Table 4:** Machine learning-based anomaly scores obtained using an Isolation Forest model for selected transmembrane pressure test values (TMP expressed in mmHg).

Mode	TMP_test_	Anomaly Score	Label
HD	300	−0.270	−1
HD	400	−0.270	−1
HD	500	−0.270	−1
HD	600	−0.270	−1
HDF	300	−0.179	−1
HDF	400	−0.289	−1
HDF	500	−0.289	−1
HDF	600	−0.289	−1

## Data Availability

The original contributions presented in this study are included in the article. Further inquiries can be directed to the corresponding author.

## Abbreviations

The following abbreviations are used in this manuscript:

TMP	Transmembrane Pressure
HD	Haemodialysis
HDF	Haemodiafiltration
EVT	Extreme Value Theory
GPD	Generalized Pareto Distribution
PDF	Probability Density Function
ML	Machine Learning
IF	Isolation Forest
IQR	Interquartile Range
